# P-377. Efficacy and Safety by Age After Switch to Doravirine/Islatravir (100 mg/0.25 mg) Once Daily: Week 48 Results from Two Phase 3 Randomized, Active-Controlled Studies in Adults Living with HIV-1

**DOI:** 10.1093/ofid/ofaf695.595

**Published:** 2026-01-11

**Authors:** Pablo Tebas, Frank A Post, Moti Ramgopal, Marcel Stoeckle, Andrew Carr, Olayemi O Osiyemi, Ronald G Nahass, Harold P Katner, Jason Szabo, Anjana Grandhi, Monica Fuszard, Stephanie O Klopfer, Rima Lahoulou, Luisa M Stamm, Michelle C Fox, Jason Y Kim

**Affiliations:** University of Pennsylvania, Merion Station, PA; King's College Hospital NHS Foundation Trust, London, England, United Kingdom; Midway Immunology and Research Center, Fort Pierce, FL; University Hospital Basel, University of Basel, Basel, Basel-Stadt, Switzerland; St Vincent’s Hospital, Sydney, New South Wales, Australia; Triple O Research Institute PA, West Palm Beach, Florida, USA, West Palm Beach, Florida; ID Care, Hillsborough, NJ; Mercer University School of Medicine, Macon, Georgia; L'Actuel Medical Clinic, Montreal, Quebec, Canada; Merck & Co., Inc., Rahway, New Jersey; Merck & Co., Inc, Rahway, New Jersey; Merck & Co., Inc., Rahway, New Jersey; MSD, Puteaux, Haute-Normandie, France; Merck & Co., Inc., Rahway, New Jersey; Merck & Co., Inc., Rahway, New Jersey; Merck & Co., Inc., Rahway, New Jersey

## Abstract

**Background:**

Doravirine/islatravir (DOR/ISL, 100 mg/0.25 mg) is an investigational once-daily regimen for HIV treatment. In two Phase 3 studies, switching to DOR/ISL was non-inferior for efficacy with a safety profile comparable to continuing baseline antiretroviral therapy (bART) or bictegravir/emtricitabine/tenofovir alafenamide (BIC/FTC/TAF) at Week 48 (W48). Because concerns about comorbidities and drug tolerability increase with age, this subgroup analysis evaluated the efficacy and safety of DOR/ISL by age.

**Figure:**
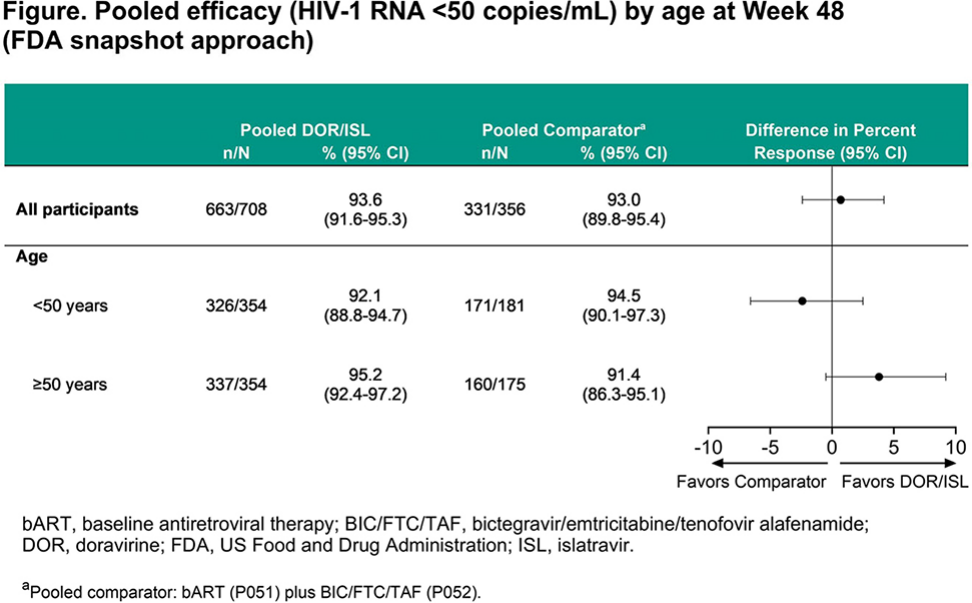

**Figure:**
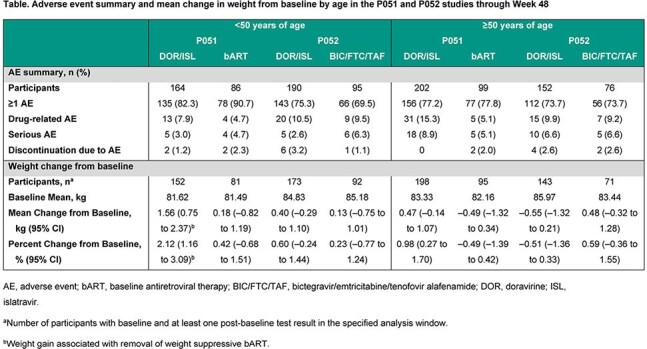

**Methods:**

Adults with HIV-1 RNA < 50 copies/mL receiving stable oral bART (MK-8591A-051 [P051]; NCT05631093) or BIC/FTC/TAF (MK-8591A-052 [P052]; NCT05630755) for ≥3 months were randomized (2:1) to switch to DOR/ISL (100 mg/0.25 mg) or to continue bART (P051) or BIC/FTC/TAF (P052). For P051+P052, efficacy results were pooled for both the DOR/ISL arms and the comparator arms (pooled comparator) and were summarized by age (< 50, ≥50 years) through W48. AEs and weight were reported by age subgroup for each study.

**Results:**

Across both studies, 708 participants switched to DOR/ISL and 356 continued bART or BIC/FTC/TAF (pooled comparator); overall 49.7% were ≥50 years old (10.6% ≥65 years). At W48, the proportion of DOR/ISL participants with HIV-1 RNA < 50 copies/mL was similar between those < 50 and ≥50 years in the pooled DOR/ISL group (92.1% vs 95.2%) and similar to the pooled comparator (94.5% vs 91.4%; Figure). Among DOR/ISL participants, rates of AEs, drug-related AEs, serious AEs, and discontinuations due to AEs were similar between age subgroups in both studies (Table). The AE profile was similar by treatment group for participants ≥65 years old. Rates of drug-related AEs were higher for open-label DOR/ISL than bART (P051), with a greater difference for participants ≥50 years, but were comparable between blinded treatment groups (P052). For DOR/ISL participants, weight changes were minimal overall (range of mean percent change in age subgroups −0.51% to 2.12%; Table). There were no clinically meaningful differences in weight change between those < 50 years and those ≥50 years.

**Conclusion:**

At W48, switching to DOR/ISL (100 mg/0.25 mg) demonstrated high efficacy and was generally well tolerated across age subgroups in adults living with HIV-1, including those ≥50 years.

**Disclosures:**

Pablo Tebas, MD, Merck: Honoraria|Shionogi: Honoraria|Viiv: Honoraria Frank A. Post, MD, PhD, Gilead: Grant/Research Support|Gilead: Honoraria|MSD: Grant/Research Support|MSD: Honoraria Moti Ramgopal, MD, FACP, FIDSA, AbbVie: Speaker Bureau|Gilead: Advisor/Consultant|Gilead: Honoraria|Shionogi Inc: Consultant|ViiV Healthcare: Advisor/Consultant|ViiV Healthcare: Honoraria Marcel Stoeckle, MD, Gilead: Advisor/Consultant|Gilead: Congress grants Andrew Carr, MD, Gilead: Board Member|Gilead: Grant/Research Support|Gilead: Honoraria|MSD: Board Member|MSD: Grant/Research Support|MSD: Honoraria|ViiV Healthcare: Board Member|ViiV Healthcare: Grant/Research Support|ViiV Healthcare: Honoraria Olayemi O. Osiyemi, MD, Gilead Sciences, Inc.: Advisor/Consultant|Gilead Sciences, Inc.: Honoraria|Merck: Advisor/Consultant|Merck: Honoraria|ViiV: Advisor/Consultant|ViiV: Honoraria Ronald G. Nahass, MD, Arbutus Pharma: Grant/Research Support|Insemed: Grant/Research Support|Janssen: Grant/Research Support|Merck: Grant/Research Support|VIR Pharma: Grant/Research Support Jason Szabo, MD, PhD, Gilead: Grant/Research Support|Gilead: Advisory board; Speaker fees|Merck: Speaker Fees|Novo Nordisk: Advisory board|ViiV: Grant/Research Support|ViiV: Advisory board; Speaker fees Anjana Grandhi, PhD, Merck & Co., Inc.: Employment|Merck & Co., Inc.: Stocks/Bonds (Public Company) Monica Fuszard, MS, Merck & Co.: Employment Stephanie O. Klopfer, PhD, Merck & Co., Inc: Employment|Merck & Co., Inc: Stocks/Bonds (Public Company) Rima Lahoulou, n/a, MSD: Employment|MSD: Stocks/Bonds (Private Company) Luisa M. Stamm, MD, PhD, Merck & Co., Inc.: Employment|Merck & Co., Inc.: Stocks/Bonds (Public Company) Michelle C. Fox, MD, Merck & Co., Inc.: Employment|Merck & Co., Inc.: Stocks/Bonds (Public Company) Jason Y. Kim, MD, MSCE, Merck & Co.: Employment|Merck & Co.: Stocks/Bonds (Public Company)

